# The association between cognitive disengagement syndrome and language in children with attention-deficit/hyperactivity disorder

**DOI:** 10.3389/fpsyt.2026.1965712

**Published:** 2026-10-19

**Authors:** Ida Jepsen, Cecilia Brynskov, Charlotte Ulrikka Rask, Kristine Jensen de López, Per Hove Thomsen, Rikke Lambek

**Affiliations:** 1 Department of Psychology and Behavioural Sciences, Aarhus University, Aarhus, Denmark; 2 Research Unit, Department of Child and Adolescent Psychiatry, Aarhus University Hospital, Aarhus, Denmark; 3 Department of Nordic Studies and Linguistics, University of Copenhagen, Copenhagen, Denmark; 4 Department of Clinical Medicine, Aarhus University, Aarhus, Denmark; 5 Department of Culture and Communication, Aalborg University, Aalborg, Denmark; 6 Department of Psychology, Agder University, Kristiansand, Norway

**Keywords:** ADHD, CDS, communication, language, pragmatics

## Abstract

**Aim:**

This study aims to explore the association between cognitive disengagement syndrome (CDS) and three aspects of language (narrative coherence, core language, and pragmatic language) in children with ADHD, and to examine whether these associations differ from those observed in neurotypical children.

**Methods:**

In this cross-sectional study, parents completed questionnaires about their child’s CDS and pragmatic language, while children with ADHD (n = 45) and neurotypical children (n = 38), aged 7–11 years, completed tasks of narrative coherence and core language.

**Results:**

CDS, after adjusting for age and sex, was significantly associated with pragmatic language in children with ADHD with a large effect (*β* = -0.51, *p* = .001), while CDS was significantly associated with core language in neurotypical children with a medium effect (*β* = -0.34, *p* = .04). There was a significant group difference in the association between CDS and pragmatic language (χ²(1) = 4.44, *p* = .04), with a stronger association in the ADHD group. After performing sensitivity analyses including inattention as an additional covariate, the associations ceased to be significant in both groups and there were no longer significant between-group differences.

**Conclusion:**

This study examined the association between CDS and language in children with and without ADHD. The findings suggest that there may be an association between CDS and pragmatic language in children with ADHD. However, sensitivity analyses including inattention as a covariate suggest that this association may largely reflect variance shared with inattention. Consequently, the association between CDS and language may partly reflect their shared association with ADHD symptoms. As this, to our knowledge, is the first study to investigate language in relation to CDS in children with ADHD, further research is warranted.

## 1 Introduction

Cognitive Disengagement Syndrome (CDS; previously named sluggish cognitive tempo) is a term applied to describe a cluster of symptoms including hypoactivity, slowed processing speed, excessive daydreaming, and under-arousal (1, 2). Symptoms of CDS are highly prevalent (25-40%) in children with Attention-Deficit/Hyperactivity Disorder (ADHD) and appear particularly associated with the ADHD inattention symptom dimension (3–5). However, current research suggests that CDS should be considered as distinct from ADHD (1, 6–8), and several studies confirm the reliability of the CDS construct (6, 7). It has been suggested that CDS and ADHD inattention can be conceptualized as two different types of attention problems, where CDS is characterized by internal/mental distractibility, while ADHD inattention is characterized by external distractibility (9). It remains unclear whether CDS should be considered a transdiagnostic factor [see e.g (10)] or be included as a specifier in the ADHD diagnostic criteria (1, 5). Research into the nature of CDS suggests that above and beyond the effect of ADHD, CDS is significantly associated with functional impairment socially (e.g., lower levels of social skills, reduced sensitivity to social cues, greater social withdrawal) and academically (e.g., lower achievement, lower grades, less homework completion), as well as with sleep disturbances and mental health problems, in particular internalizing symptoms of anxiety and depression (2, 6–8). However, several aspects of the syndrome remain underinvestigated, for example Becker et al. (1) have recommended that the relationship between CDS and functional impairment be examined further.

In children and adolescents with ADHD, research suggests that around 40% also fulfill criteria for a language disorder (11). Furthermore, children and adolescents with ADHD (at the group level) have a higher rate of pragmatic language difficulties (12–14) and obtain lower scores on standardized language tasks (15, 16) compared to their neurotypical peers. These language problems appear to be associated with social challenges and academic problems (16), suggesting that many children and adolescents with ADHD have functionally impairing language problems (17). While the association between ADHD and language problems is now relatively well established [e.g (12, 15, 18)], less is known about the factors associated with the co-occurrence of ADHD and language problems (17). Here, CDS may be a candidate. To date, very few studies have examined the association between CDS and language problems. Barkley (3) compared groups of children with ADHD (*n* = 95), CDS (*n* = 41), ADHD+CDS (*n* = 61), and neurotypical children (*n* = 1603) and found that the prevalence of language delay was highest in the ADHD+CDS group. A similar study was performed by Burns & Becker (19), who also found the highest prevalence of language delay in children with ADHD+CDS (*n* = 49) compared to the ADHD-only (*n* = 93), CDS only (*n* = 53), and neurotypical (*n* = 1861) groups. Russell et al. (20), in the only study to examine the association between CDS and language to date, found children with heightened CDS symptoms (*n* = 23) to have more social pragmatic language difficulties (as indicated by parent report) than children with low CDS with a medium effect. Further examining what are considered three important clinical markers of language disorder, they found a small but significant difference in sentence recall but not tense markings or nonword repetitions (20). In a similar vein, a study comparing 58 autistic children to 49 neurotypical children, found that CDS explained social pragmatic communication difficulties above and beyond that explained by core characteristics of autism, age, sex, and social economic status (21). Taken together, very preliminary research suggests a link between CDS and language delay as well as social pragmatic language problems, possibly due to CDS reducing the efficiency with which children encode, organize, and retrieve linguistic information (20, 22). To our knowledge, the association between CDS and language problems has never been investigated in children with ADHD before.

Consequently, the overall aim of the current study was, for the first time, to examine the association between CDS and three different aspects of language (pragmatic language, core structural language, and coherence in storytelling) in children with ADHD. First, we examined whether CDS and language in children ADHD are associated. Second, we examined whether potential associations between CDS and language in children with ADHD are significantly different from associations observed in neurotypical children. As the study is exploratory and previous research is very sparse (20), we have no formal hypotheses but expect to find the strongest association between CDS and pragmatic language in both groups, as was found in Russell et al. (20) and Karaca & Özyurt (21). Due to lack of previous research, we have no formal hypothesis regarding core language and narrative coherence. This study is part of a larger study about ADHD and language (16, 23). The data on CDS has not been published before.

## 2 Methods

The study is based on the data from the research project “Language in children with and without ADHD”, which was collected between May 2018 and January 2022. The project was approved by the Danish regional ethics committee (1-10-72-443-17), registered with the Danish data protection agency (2016-051-000001), and clinical trials (NCT03917316). All participating families received extensive written and verbal information, legal guardians gave verbal and written consent for child participation, and all children gave verbal assent. Below is a brief description of the participants, procedures and materials included in the current study. A detailed description can be found in Jepsen et al. (16, 23).

### 2.1 Participants

Children with ADHD (*n* = 46) were recruited from a specialist ADHD clinic at a Danish public regional child and adolescent psychiatric hospital, while neurotypical children (NC, *n* = 40) were recruited from schools in the same region as the hospital. Inclusion criteria for both groups included age between 7 and 11 years, 11 months and 30 days, Danish as a first language, no history of autism, epilepsy, or brain damage, IQ ≥ 70, birth after the 32. gestation week and birth weight ≥ 1500 gr., as well as sight, hearing, and motor function at a level where the child could complete the tasks. For the ADHD group specifically, inclusion criteria were an (ICD-10) ADHD diagnosis based on assessment at the specialist ADHD clinic, and in case they received psychotropic medication (*n* = 22), this had to be discontinued >24h before assessment. Participants were included in the present study if their parents had completed the CDS questionnaire, which resulted in a final sample of 45 children with ADHD and 38 NCs. See **Table 1** for the characteristics of the final sample.

**Table 1:** Sample characteristics.

Characteristic	ADHD (*n* = 45)	NC (*n* = 38)	*t* or *x^2^*	*p*	Effect size^c^
Age (*Mean* [SD])	9.69 (1.19)	9.17 (1.41)	-1.85	.07	.41
Sex (*n*) Boys Girls	37 8	16 22	12.64	<.001	.39
Raven’s CPM standard score (*Mean* [*SD*])^a^ Co-occurring ICD-10 diagnoses (*n*) F81-89 (excluding F84) F95x Other behavioral and/or emo.dis. F85 Tic disorder	100.68 (16.83) 18 ≤5 ≤5	110.92 (16.14) - - -	2.80 - - -	.006 - - -	.62 - - -
Parental education in years (*Mean* [*SD*])^b^	13.95 (2.30)	16.39 (1.54)	5.41	<.001	1.22

CPM, Colored Progressive Matrices; ICD-10, Internal Classification of Diseases-10; NC, neurotypical comparison children; SD, standard deviation; emo, emotional; dis, disorder.

^a^ ADHD = 44, NC = 38.

^b^ ADHD = 43, NC = 36.

^c^ Cohen’s *d* or Phi. Cohen’s *d* small effect = .20, medium effect = .50, large effect ≥.80 (Cohen, 1988); Phi small effect = 0.1, medium effect = 0.3, large effect ≥0.5 (Field, 2007).

### 2.2 Procedures

Language and cognitive assessments were carried out in a quiet room at Aarhus University during two separate sessions of approximately 1.5 hours each. Parents (or legal guardians) received an extensive questionnaire battery, including questions about CDS, pragmatic language, and inattention.

### 2.3 Materials

*Cognitive Disengagement Syndrome (CDS)* was measured using three parent-rated items from the Strengths and Weaknesses of Attention-Deficit/Hyperactivity-Disorder symptoms and Normal behavior Scale (SWAN) (24). The items address three core aspects of CDS: *daydreaming/lost in thought*, *drowsiness/lack of energy* and *apathetic/unmotivated* (2, 24). Parents were asked to compare their child to peers on a 7-point Likert scale from 3 (far below average) to -3 (far above average). The CDS outcome was the mean of the three items, with higher scores indicating higher rates of CDS. This outcome has previously been found to have acceptable internal consistency (α =.73; (25)). In the current study, the CDS composite also had acceptable internal consistency (McDonald’s omega ADHD α = .78, NC α = .89), and acceptable interrater reliability with teacher ratings in both groups (ADHD *n* = 25, *r* = .63, NC *n* = 17, *r* = .61).

*Narrative coherence* was examined using “Frog, where are you?” (26), a wordless picture book (see 23, for transcription and coding procedures). In the task, the child was encouraged to tell the story depicted in the book using their own words while looking through it. A narrative coherence score was calculated by summing ratings of central story elements (i.e., beginning, middle, and end) with higher scores indicating more coherence. The ratings were performed by trained, independent coders, who were blind to child diagnostic status. Excellent reliability of coding (α = .96) was found for this outcome (see 23).

*Core language* was examined using the tasks from the Core language Index in the Clinical Evaluation of Language Fundamentals-4 (CELF-4) (27). The tasks included Concepts and following directions, Formulating Sentences, Word Classes 1 (<9 y.o.), Word Classes 2 (>9 y.o.), and Sentence Structure (<9 y.o.). Higher scaled scores indicated better core language skills. The Scandinavian version of these subtests has been found to have acceptable to excellent internal consistency (range α = .78-.94) (27).

*Pragmatic language* was examined using Inappropriate Initiations, Stereotypical Language Use, Use of Conversational Context, and Non-verbal Communication subscales from the parent version of the Children’s Communication Checklist-2 (CCC-2) (28). Each subscale contained seven items rated on a 4-point Likert scale (28). The four total subscale scores were converted to age normed scaled scores and summed (in line with (29)) with higher scores indicating better pragmatic language skills. Good internal consistency for the pragmatic language composite was previously found in the current sample (ADHD α = .86, NC α = .89; see 16) and the composite had acceptable interrater reliability with teacher ratings in the ADHD group (*n* = 24, *r* = .60) but not in the NC group (*n* = 17, *r* = .17).

*Inattention* was measured based on nine ADHD inattention items from the SWAN (24). Parents were asked to compare their child to peers on a 7-point Likert scale from 3 (far below average) to -3 (far above average). The inattention outcome was the mean of the 9 items, with higher scores indicating higher levels of inattention. Good to excellent internal consistency for this outcome was found in the current sample (ADHD α = .86, NC α = .90), and good interrater reliability with teacher ratings was previously established in this sample (ADHD, n = 25, *r* = .57, NC n = 20, *r* = .61) (23).

### 2.4 Statistical analysis

Missing cases ranged from 0% to 2.3%. There were no missing items. Visual inspection of data indicated no extreme outliers. A multiple-group multivariate regression model was performed to examine associations between CDS and the three language outcomes (narrative cohesion, core language, and pragmatic language). Models were estimated using maximum likelihood estimation with robust standard errors (MLR), with FIML-type handling of missing data. The three language outcomes were simultaneously regressed on CDS with the residual variances of the three outcomes allowed to covary. Regression coefficients were estimated for children with ADHD and neurotypical children while controlling for age and sex (boy = 0, girls = 1). Wald χ² tests were used to evaluate group differences in CDS–outcome associations, first jointly across all outcomes and then separately for each regression coefficient. Given the potential overlap between CDS and ADHD inattention symptoms (3, 4), a sensitivity analysis controlling for inattention was also conducted. General cognitive functioning was not controlled for in the analyses as this may eliminate variance due to ADHD (30) and produce overcorrected results (31). The analyses were performed in MPLUS Version 8.11 (32).

## 3 Results

Descriptive statistics for the study variables are presented in **Table 2**, while bivariate correlations can be found in the **Supplementary Materials** (**Supplementary Tables S1**-**S3**).

**Table 2:** Descriptives.

Measure	ADHD (*n* = 45) Mean (SD) Range		NC (*n* = 38) Mean (SD) Range		*t*	*p*	Cohen’s *d*
Narrative coherence total score^a^	4.02 (1.19)	1-6	4.79 (1.12)	2-6	2.99	.004	0.66
CELF-4 Core language sum score	22.76 (7.05)	7-37	30.37 (6.52)	15-40	5.07	<.001	1.12
CCC-2 Pragmatic language sum score	23.71 (8.71)	10-59	44.71 (8.48)	25-55	11.08	<.001	2.44
CDS mean score	1.05 (0.93)	(-1)-3	-0.80 (1.02)	(-3)-0.67	-8.66	<.001	1.91

CCC-2, Childrens Communicaton Checklist-2; CDS, Cognitive Disengagement Syndrome; CELF-4, Clinical Evaluation of Language Fundamentals-4; NC, neurotypical comparison children; SD, standard deviation.

^a^ ADHD = 44, NC = 38.

The results from the multiple-group multivariate regression model are presented in **Table 3** (ADHD group) and **Table 4** (NC group). In the ADHD group, CDS (adjusting for age and sex), explained a statistically significant amount of variance in pragmatic language, indicating that the children with ADHD with higher parent rated CDS also had lower parent rated pragmatic language skills (*β* = -0.51, *SE* = 0.10, *p* = .001, 95% CI for *β* [-0.71, -0.32]), representing a large effect. CDS was not significantly associated with narrative coherence or core language in the ADHD group (both *p*s >.05) and the effect sizes were small (both *β*s ≤ 0.26). In the NC group, CDS, again adjusting for age and sex, explained a significant amount of the variance in core language, indicating that neurotypical children with higher parent rated CDS also had lower core language scores (*β* = -0.34, *SE* = 0.16, *p=*0.04, 95% CI for *β* [-0.65, -0.04]), representing a medium effect. There were no significant associations between CDS and narrative coherence or pragmatic language in the NC group (both ps > 0.05) and the effect sizes were very small (both *β*s *≤-*0.13).

**Table 3:** Multivariate regression analysis of the association between CDS and coherence in narrative language, core structural language abilities, and pragmatic language controlling for age and sex in the ADHD group.

Dependent variable	Independent variable	Unstand.					Stand.	
		B	*CI^a^*	*S.E.*	*z*	*p^b^*	*β^c^*	*R^2^*
Narrative Coherence	CDS	0.290	[-0.04, 0.62]	0.167	1.736	.083	0.226	.102
	Sex	0.448	[-0.26, 1.15]	0.360	1.244	.213	0.152	
	Age	0.095	[-0.20, 0.39]	0.151	0.633	.527	0.095	
Core language	CDS	-1.984	[-4.40, 0.43]	1.231	-1.612	.107	-0.262	.089
	Sex	2.425	[-1.43, 6.28]	1.965	1.235	.217	0.139	
	Age	1.295	[-0.72, 3.31]	1.026	1.263	.207	0.218	
Pragmatic language	CDS	-4.824	[-7.65, -2.00]	1.441	-3.348	.001	-0.514	.264**
	Sex	4.009	[-1.37, 9.38]	2.742	1.462	.144	0.186	
	Age	-0.056	[-1.98, 1.86]	0.980	-0.057	.954	-0.008	

CDS, Cognitive Disengagement Syndrome; CI, Confidence Intervals; SE, standard error; stand., standardized; unstand., unstandardized.

*^a^95%* CIs for unstandardized B-values.

*^b^p* based on unstandardized values.

^c^yx-standardized.

^**^p <.01.

**Table 4:** Multivariate regression analysis of the association between CDS and coherence in narrative language, core structural language abilities, and pragmatic language controlling for age and sex in the neurotypical group.

Dependent variable	Independent variable	Unstand.					Stand.	
		B	*CI^a^*	*S.E.*	*z*	*p^b^*	*β^c^*	*R^2^*
Narrative Coherence	CDS	-0.144	[-0.44, 0.15]	0.152	-0.945	.345	-0.130	.287*
	Sex	0.593	[-0.01, 1.19]	0.307	1.932	.053	0.265	
	Age	0.404	[0.19, 0.62]	0.107	3.762	.000	0.508	
Core language	CDS	-2.211	[-4.35, -0.07]	1.090	-2.027	.043	-0.344	.156
	Sex	2.294	[-1.73, 6.31]	2.051	1.118	.264	0.176	
	Age	0.104	[-1.31, 1.52]	0.722	0.144	.886	0.022	
Pragmatic language	CDS	-0.542	[-3.35, 2.26]	1.432	-0.379	.705	-0.065	.058
	Sex	-3.947	[-8.69, 0.80]	2.421	-1.631	.103	-0.233	
	Age	-0.616	[-2.44, 1.21]	0.931	-0.661	.508	-0.102	

CDS, Cognitive Disengagement Syndrome; CI, Confidence Intervals; SE, standard error; stand., standardized; unstand., unstandardized.

*^a^*95% CIs for unstandardized B-values.

*^b^p* based on unstandardized values.

^c^yx-standardized.

^*^p <.05.

The omnibus Wald chi-square test was significant (*X^2^*(3) = 12.06, *p* = .007), indicating overall significant differences between the two groups. The individual Wald Chi-square tests indicated that there was a significant difference between the slopes of the association between CDS and pragmatic language (*X^2^*(1) = 4.44, *p* = .04), with a stronger association between CDS and pragmatic language in the ADHD group compared to the NC group. The difference between the slopes for the associations between CDS and narrative language was not statistically significant (*X^2^*(1) = 3.68, *p* = .055), and neither were the slopes for the association between CDS and core language, (*X^2^*(1) = 0.02, *p* = .89).

After the inclusion of ADHD inattention as a covariate in the model (see **Supplementary Tables S4**, **S5** in the **Supplementary Material**), the association between CDS and pragmatic language in the ADHD group [*β* = -0.26, SE = 0.13, *p* = .08, 95% CI for *β* (-0.50, -0.01)], and the association between CDS and core language in the NC group [*β* = -0.20, SE = 0.25, *p* = .44, 95% CI for *β* (-0.70, 0.30)] were no longer statistically significant and effects were small. The omnibus Wald test remained significant (*X^2^*(3) = 9.02, *p* = .03). However, individual Wald chi-square tests comparing slopes suggested that the difference between the groups on the association between CDS and pragmatic language was no longer statistically significant [*X^2^*(1) = 2.07, *p* = .15].

## 4 Discussion

The current study investigated the association between CDS and language in children with ADHD. We found support for an association between CDS and pragmatic language in children with ADHD and this association was significantly stronger compared to the group of neurotypical children. After including inattention as a covariate, the association was no longer significant, suggesting that the association between CDS and pragmatic language may partly reflect variance shared with inattention. Given the exploratory nature of this study, the findings should be considered preliminary and require replication in larger, independent samples.

In line with our predictions, CDS was significantly associated with pragmatic language in children with ADHD. This finding is on par with previous results reported in a sample of autistic children (21) and neurotypical children (20). Further, the association was significantly stronger in children with ADHD compared to the neurotypical control group, suggesting that the relationship between CDS and language differs between the groups. Pragmatic language can, broadly, be defined as the verbal and nonverbal aspects of understanding and contributing to social interactions (12, 33, 34), while the subdomains of the CCC-2 used in the present study, specifically, target discourse management behaviors, i.e., verbal and nonverbal aspects of initiating, maintaining, and ending conversations (28, 35). For children with ADHD, it may be that daydreaming, lack of motivation, and/or slower cognitive processing challenges the ability to detect and react to subtle cues during conversation, resulting in less initiative in as well as less attention to the conversational context. If this is the case, difficulties in pragmatic language may also be involved in the social challenges found to be associated with CDS such as struggling with social skills and reduced sensitivity to social cues (2, 6–8), and could require targeted support when working with children with ADHD and CDS. Subsequent sensitivity analyses indicated that the association between CDS and pragmatic language in the ADHD group was no longer significant after inclusion of inattention as an additional covariate (*p* = .08), just as the between-group difference ceased to be significant once inattention was included in the model [*p* = (15)]. Among children with ADHD, inattention was significantly associated with pragmatic language difficulties (see **Supplementary Table S4**), suggesting that the observed relationship between CDS and pragmatic language problems may largely reflect variance shared with inattention. Though research has found CDS and inattention to be distinct constructs (see 6,7), considerable overlap between the two is also observed [see e.g (4)], and there is currently no consensus regarding how to deal with this issue. Consequently, studies differ with regards to whether they correct [see e.g (10, 36, 37)] or do not correct for inattention when examining CDS [see e.g (3, 19–21, 38)]. Controlling for inattention may be meaningful as inattention is independently associated with language problems [see (14, 39)]. However, because CDS and inattention share substantial variance, such adjustment may also remove variance that is relevant to the CDS construct, potentially resulting in overadjustment (4), much like the practice of controlling for IQ in ADHD research (29). Neither Russell et al. (20) nor Karaca & Özyurt (21) controlled for inattention, so it is not possible to determine whether their results would have changed had inattention been included as a covariate. Presently, it is unclear whether the sensitivity analyses in the current study removed variance that is relevant to the CDS construct or suggest that the association between CDS and language is largely attributable to variance shared with inattention. As this, to our knowledge, is the only study to date to investigate the association between CDS and language in children with ADHD, further research is needed to determine whether CDS is specifically associated with pragmatic language problems in children with ADHD. In the future, this might be accomplished by using methods such as structural equation modelling to establish the discriminant validity of the two constructs (CDS and inattention) and to determine the amount of unique variance in pragmatic language explained by each construct. Clarification of the nature of the relationship between CDS, inattention, and pragmatic language has important clinical implications, for instance, as pragmatic language difficulties appear to negatively impact social skills in children with ADHD [see e.g (16)], children with ADHD and pragmatic language problems may benefit from support programs targeting CDS and/or inattention. Further, language problems in children with ADHD appear under-identified [see e.g (11)], and if a specific association between CDS and pragmatic language is supported, the presence of CDS could possibly prompt the clinician to screen for pragmatic language problems during clinical assessment for ADHD – and vice versa.

We found a significant association between CDS and core language in neurotypical children. However, the between-group difference in the association was not significant, and in sensitivity analyses including inattention as a covariate, the association between CDS and core language in neurotypical children ceased to be significant. The association between CDS and core language was not significant in the ADHD group. Core language captures formal aspects of language closely associated with language proficiency and builds on rule-based knowledge, including being able to produce and understand complex sentences due to implicit knowledge about syntactic and grammatical rules such as word order and tense. Core language abilities also involve being able to categorize, understand, and use overarching and abstract concepts such as “feelings” or “colors” (27), and children with ADHD are consistently found to perform below their neurotypical peers on standardized core language tasks [see e.g (15)]. It is possible that for children with ADHD, other aspects - e.g., co-occurring language disorder – have a more important impact on their core language abilities than CDS. Regarding the ability to produce coherent stories, the current study did not support an association between CDS and narrative coherence in children with or without ADHD, nor did it support a significant between-group difference regarding this association, and sensitivity analyses including inattention as a covariate did not change this result. Thus, the current study does not support a specific association between CDS and narrative coherence in children with ADHD.

To our knowledge, the current study is the first investigation of the potential association between CDS and language in children with ADHD specifically. The strengths include a larger sample size compared to the previous (and very few) studies on language and CDS, the examination of several different language aspects, the investigation of the association in two different groups (ADHD and neurotypical), and inclusion of sensitivity analyses controlling for inattention. The study is, however, considered exploratory and has important limitations. First, due to the relatively small sample size, the study likely has limited power to detect smaller associations or subtle between-group differences. Therefore, we remain cautious in our interpretations of the findings, and recommend that the results are replicated in larger, independent samples. Second, as the study is cross-sectional, we cannot detangle causal relationships between variables, which should be investigated in longitudinal studies. For example, symptoms of CDS may be more apparent in older children and adolescents either due to symptoms worsening or changing environmental expectations (1), and thus investigating the association between ADHD, CDS, and language development over time and including older age groups might provide important information about the impact of CDS on language development in children with ADHD. Third, CDS, inattention, and pragmatic language were all rated by parents, and some of the associations may reflect same-rater bias. Due to the Covid-19 pandemic, only a proportion of teachers returned questionnaires (e.g., only 50-56% of teachers completed the CDS items), and as a result, teacher ratings were only included to assess inter rater reliability of the CDS, inattention, and pragmatic language measures in the present study. While the inter-rater agreement was generally good, suggesting that parent ratings provided a reliable representation of the constructs across informants, future studies should examine CDS, inattention, and pragmatic language using a multi-informant design, observations, or performance-based measures. Fourth, there was a significant difference between the groups regarding sex distribution. While we corrected for sex in the analyses, it is important to include more girls with ADHD in future research regarding CDS and language to examine if the findings are generalizable to girls as well as boys [see e.g (40)]. Finally, we did not control for internalizing symptoms in the present study. Previous research suggests that internalizing symptoms are associated with language as well as CDS [see e.g (20)]. In the current study, five or fewer participants in the ADHD group had a co-occurring emotional disorder (see **Table 1**). However, in future studies, the effect of internalizing symptoms is important to examine when investigating the association between CDS and language.

Taken together, the current study suggests that there may be an association between CDS and pragmatic language in children with ADHD. However, sensitivity analyses including inattention as a covariate suggest that the association between CDS and pragmatic language may largely reflect their shared association with inattention. Further, the current study does not find support for an association between CDS and core language or narrative coherence in children with ADHD. As this, to our knowledge, is the only study to date to investigate these aspects of language in relation to CDS in children with ADHD, the results need to be replicated and further examined in larger samples using research designs that can help determine whether the association between CDS and pragmatic language in children with ADHD is largely attributable to variance shared with inattention or not. In the future, understanding the relationship between ADHD, CDS, and pragmatic language may have important clinical implications, particularly for supporting the social challenges often associated with ADHD and co-occurring pragmatic language problems [see e.g (16)].

## Data Availability

The datasets presented in this article are not readily available due to legal restrictions. Requests to access the datasets should be directed to ibj@psy.au.dk.
