# Supplementary material for: The association between cognitive disengagement syndrome and language in children with attention-deficit/hyperactivity disorder
Source: Front Psychiatry. 2026 Oct 19;17:1965712. doi: 10.3389/fpsyt.2026.1965712 (PMC13725549; doi:10.3389/fpsyt.2026.1965712)
Supplement: Supplementary file 1 [file Supplementaryfile1.pdf]

Supplementary Material

Table S1 Correlations[a] between CDS and language without adjusting for age and sex (whole sample; n = 83)

	CDS	Pragmatic language	Coherence	Core language
CDS	1			
Pragmatic language	-.665***	1		
Coherence	-.155	.315**	1	
Core language	-.507***	.563***	.248*	1

Note. CDS = Cognitive Disengagement Syndrome.

[a]Pearson r.

*p<0.05, **p<.01, ***p<.001

Table S2 Correlations[a] between CDS and language without adjusting for age and sex (ADHD group; n = 45)

	CDS	Pragmatic language	Coherence	Core language
CDS	1			
Pragmatic language	-.480***	1		
Coherence	.276	.185	1	
Core language	-.189	.484***	.010	1

Note. CDS = Cognitive Disengagement Syndrome.

[a]Pearson r. ***p<.001

Table S3 Correlations[a] between CDS and language without adjusting for age and sex (NC group; n = 38)

	CDS	Pragmatic language	Coherence	Core language
CDS	1			
Pragmatic language	-.055	1		
Coherence	-.112	.053	1	
Core language	-.356*	.173	.245	1

Note. CDS = Cognitive Disengagement Syndrome.

[a]Pearson r.

*p<.05

Table S4. Multivariate regression analysis of the association between CDS and coherence in narrative language, core structural language abilities, and pragmatic language controlling for age, sex, and inattention in the ADHD group

Dependent variable	Independent variable	Unstand.					Stand.	
		B	*CI*[a]	*S.E.*	*z*	*p*[b]	β^c	R^2
Narrative Coherence	CDS	0.421	[-0.05, 0.89]	0.238	1.769	.077	0.329	.120
	Sex	0.471	[-0.23, 1.17]	0.355	1.327	.185	0.160	
	Age	0.101	[-0.20, 0.40]	0.151	0.669	.504	0.101	
	Inattention	-0.275	[-0.77, 0.22]	0.253	-1.086	.278	-0.172	
Core language	CDS	-1.136	[-4.18, 1.91]	1.555	-0.731	.465	-0.150	.110
	Sex	2.570	[-1.53, 6.67]	2.091	1.229	.219	0.148	
	Age	1.330	[-0.66, 3.32]	1.016	1.309	.191	0.224	
	Inattention	-1.768	[-4.99, 1.45]	1.643	-1.076	.282	-0.186	
Pragmatic language	CDS	-2.412	[-5.07, 0.25]	1.358	-1.777	.076	-0.257	.379***
	Sex	4.421	[-0.65, 9.50]	2.589	1.708	.088	0.205	
	Age	0.045	[-1.77, 1.86]	0.925	0.049	.961	0.006	
	Inattention	-5.033	[-8.08, -1.98]	1.557	-3.234	.001	-0.429	

Note. CDS = Cognitive Disengagement Syndrome, CI = Confidence Intervals, SE = standard error, stand. = standardized, unstand. = unstandardized.

[a] *95%* CIs for unstandardized B values

[b] *p* based on unstandardized values

[c] yx-standardized

*** $p<.001$

Table S5. Multivariate regression analysis of the association between CDS and coherence in narrative language, core structural language abilities, and pragmatic language controlling for age, sex, and inattention in the NC group

Dependent variable	Independent variable	Unstand.					Stand.	
		B	*CI*[a]	*S.E.*	*z*	*p*[b]	*β*[c]	R^2
Narrative Coherence	CDS	-0.180	[-0.57, 0.21]	0.198	-0.910	.363	-0.163	.289*
	Sex	0.603	[0.02, 1.19]	0.299	2.014	.044	0.270	
	Age	0.404	[0.19, 0.61]	0.107	3.766	.000	0.507	
	Inattention	0.060	[-0.29, 0.41]	0.179	0.338	.735	0.048	
Core language	CDS	-1.281	[-4.52, 1.96]	1.652	-0.776	.438	-0.199	.180*
	Sex	2.032	[-1.73, 5.80]	1.921	1.057	.290	0.156	
	Age	0.107	[-1.30, 1.52]	0.718	0.150	.881	0.023	
	Inattention	-1.553	[-4.56, 1.45]	1.533	-1.013	.311	-0.213	
Pragmatic language	CDS	0.733	[-2.63, 4.09]	1.713	0.428	.669	0.088	.084
	Sex	-4.306	[-8.99, 0.37]	2.387	-1.804	.071	-0.254	
	Age	-0.611	[-2.46, 1.24]	0.943	-0.648	.517	-0.101	
	Inattention	-2.132	[-5.60, 1.34]	1.770	-1.205	.228	-0.224	

Note. CDS = Cognitive Disengagement Syndrome, CI = Confidence Intervals, SE = standard error, stand. = standardized, unstand. = unstandardized.

[a]*95%* CIs for unstandardized B values

[b]*p* based on unstandardized values

[c]yx-standardized

$^*p<.01$

frontiers
